# The effects of multiple chronic conditions on hospitalization costs and utilization for ambulatory care sensitive conditions in the United States: a nationally representative cross-sectional study

**DOI:** 10.1186/s12913-016-1304-y

**Published:** 2016-03-01

**Authors:** Halcyon G. Skinner, Rosanna Coffey, Jenna Jones, Kevin C. Heslin, Ernest Moy

**Affiliations:** Truven Health Analytics, 4819 Emperor Blvd., Suite 125, Durham, NC 27703 USA; Truven Health Analytics, 7700 Old Georgetown Rd., Bethesda, MD 20814 USA; Agency for Healthcare Research and Quality, 5600 Fishers Lane, Rockville, MD 20857 USA

**Keywords:** Multiple chronic conditions, Multimorbidity, Claims data, Inpatient hospitalization, Hospital costs, Ambulatory care sensitive conditions

## Abstract

**Background:**

The presence of multiple chronic conditions (MCCs) complicates inpatient hospital care, leading to higher costs and utilization. Multimorbidity also complicates primary care, increasing the likelihood of hospitalization for ambulatory care sensitive conditions. The purpose of this study was to evaluate how MCCs relate to inpatient hospitalization costs and utilization for ambulatory care sensitive conditions.

**Methods:**

The 2012 Agency for Healthcare Research and Quality (AHRQ) Healthcare Cost and Utilization Project (HCUP) State Inpatient Databases (SID) provided data to carry out a cross-sectional analysis of 1.43 million claims related to potentially preventable hospitalizations classified by the AHRQ Prevention Quality Indicator (PQI) composites. Categories of MCCs (0–1, 2–3, 4–5, and 6+) were examined in sets of acute, chronic, and overall PQIs. Multivariate models determined associations between categories of MCCs and 1) inpatient costs per stay, 2) inpatient costs per day, and 3) length of inpatient hospitalization. Negative binomial was used to model costs per stay and costs per day.

**Results:**

The most common category observed was 2 or 3 chronic conditions (37.8 % of patients), followed by 4 or 5 chronic conditions (30.1 % of patients) and by 6+ chronic conditions (10.1 %). Compared with costs for patients with 0 or 1 chronic condition, hospitalization costs per stay for overall ambulatory care sensitive conditions were 19 % higher for those with 2 or 3 (95 % confidence interval [CI] 1.19–1.20), 32 % higher for those with 4 or 5 (95 % CI 1.31–1.32), and 31 % higher (95 % CI 1.30–3.32) for those with 6+ conditions. Acute condition stays were 11 % longer when 2 or 3 chronic conditions were present (95 % CI 1.11–1.12), 21 % longer when 4 or 5 were present (95 % CI 1.20–1.22), and 27 % longer when 6+ were present (95 % CI 1.26–1.28) compared with those with 0 or 1 chronic condition. Similar results were seen within chronic conditions. Associations between MCCs and total costs were driven by longer stays among those with more chronic conditions rather than by higher costs per day.

**Conclusions:**

The presence of MCCs increased inpatient costs for ambulatory care sensitive conditions via longer hospital stays.

## Background

In international comparisons, The U.S. health care system ranks best in the provision of preventive and patient-centered care [1], but lags in efficiency and access. The top 1 % of U.S. patients’ utilization accounts for 23 % of health care expenditures [2]. Many of these high health care users are diagnosed with more than one condition and may have complicated ambulatory care needs. Among Americans, 25 % have multimorbidity, otherwise known as a multiple chronic conditions (MCC) defined as two or more concurrent chronic conditions [3], and 68–80 % of people aged 65 years or older have MCC [4, 5].

Currently, more than two-thirds of all hospital discharges in the United States are for individuals with MCCs [6]. The U.S. Department of Health and Human Services (HHS) has identified the prevalence of MCCs and associated consequences in the United States as a key health care concern [7]. Presence of MCCs has been shown to complicate inpatient hospital care [8], leading to higher costs and utilization [6, 9–11]. For example, a 1999 study of older adults in the United States found that, on average, Medicare paid over $13,000 more each year for medical care for beneficiaries with more than 3 chronic conditions, than those with none [11]. That same study found that, per capita, the cost per beneficiary with no chronic conditions was less than $1000. At the same time, multimorbidity complicates ambulatory care, which can increase the likelihood of hospitalization for potentially preventable conditions [12–14].

The U.S. Department of Health and Human Services, is focusing on people with MCCs as one target for improving care for vulnerable populations. The HHS initiative on MCCs aims to assess the burden of MCCs on the health of the population and to evaluate the role of MCCs in health care utilization, quality, and costs. The goal is to inform future health policies to improve care and reduce cost [15]. From the 2012 implementation of the HHS Strategic Framework on Multiple Chronic Conditions, HHS is focusing on dissemination of data and advancement of quality measures [7].

The Agency for Healthcare Research and Quality (AHRQ) Prevention Quality Indicators (PQIs) are a set of measures designed to quantify the occurrence of potentially preventable hospitalizations among ambulatory care sensitive conditions. These measures of population health are useful for tracking trends in hospitalization for conditions linked to the quality of ambulatory care. In all regions of the United States, hospitalizations for PQI conditions have declined in recent years [16]. Yet, the impact of multimorbidity on utilization and cost is relatively unknown. Such information could lead to improved guidelines of care that address the complexities of interacting conditions. Condition-specific data also could allow for more targeted interventions on high-cost, high-utilizing multimorbidity populations.

The purpose of this study was to evaluate how the presence of MCCs relates to inpatient hospitalization costs and utilization for sets of conditions that are potentially preventable through high-quality ambulatory care.

## Methods

### Data and study population

We used data from the 2012 AHRQ Healthcare Cost and Utilization Project (HCUP) State Inpatient Databases (SID) to carry out a cross-sectional analysis. In the SID, we identified 1.43 million discharge records, representing 3.58 million (weighted) potentially preventable hospitalizations. We selected a sub-sample of the SID, the SID disparities analysis file, which is used to compute national estimates for the National Healthcare Disparities Report. It consists of weighted records from a sample of hospitals from 38 States participating in HCUP that have high-quality race/ethnicity data in 2012: AK, AR, AZ, CA, CO, CT, FL, GA, HI, IA, IL, IN, KS, KY, MA, MD, MI, MO, NC, NJ, NM, NV, NY, OH, OK, OR, PA, RI, SC, SD, TN, TX, UT, VA, VT, WA, WI, and WY. The SID disparities analysis file contains records representing 91 % of all U.S. hospital discharges. Nationally-representative statistics were computed using discharge weights that were constructed with consideration of different attributes of the hospital, including the number of beds, geographic region, number of discharges and teaching status. We selected records for U.S. adults aged 18 years and older discharged from U.S. community, non-rehabilitation hospitals with a primary diagnosis of an ambulatory care sensitive condition contained within AHRQ PQIs. All investigators completed training and signed a data use agreement for the HCUP SID (https://www.hcup-us.ahrq.gov/tech_assist/dua.jsp). Because data for our analyses did not involve human subjects, IRB approval was not required. HCUP SID data are available via the HCUP Central Distributor (https://www.hcup-us.ahrq.gov/tech_assist/centdist.jsp).

We categorized hospitalizations into subsets based on three PQIs focused on broad composites of potentially preventable hospitalizations: the acute composite (PQI 91), the chronic composite (PQI 92), and the overall composite (PQI 90). The acute composite (PQI 91) included hospitalizations for dehydration, bacterial pneumonia, and urinary tract infection (*n* = 559,515). The chronic composite (PQI 92) included hospitalizations for diabetes (short-term complications, long-term complications, uncontrolled diabetes, and lower-extremity amputation for diabetes), chronic obstructive pulmonary disease (COPD) or asthma in older adults, hypertension, congestive heart failure, angina without a procedure, and asthma in younger adults (*n* = 866,668). The Overall Composite (PQI 90) was the union of the three PQI 91 conditions and the nine PQI 92 conditions (*n* = 1,426,153). Discharge records were scored using version 4.4 of the AHRQ Quality Indicator SAS software to identify hospital stays that fit the definitions of the PQI composites [17].

### Primary independent variable

Our primary independent variable was the number of MCCs present in primary and secondary diagnosis codes. We created categories of MCCs grouped as 0–1 condition, 2–3 conditions, 4–5 conditions, and 6+ conditions from the list of conditions defined by the HHS Strategic Framework [7, 18]. To be included in the chronic PQI sample, patients had to have at least 1 chronic condition; therefore the “0–1 condition” reference category for these patients included those with exactly 1 chronic condition, whereas patients with an acute PQI were placed in the “0–1 condition” category if they had either 0 or 1 concurrent chronic condition. The 20 chronic conditions included were arthritis, asthma, autism spectrum disorder, coronary artery disease (CAD), cancer, cardiac arrhythmias, chronic kidney disease (CKD), congestive heart failure (CHF), COPD, dementia, depression, diabetes, hepatitis, HIV, hyperlipidemia, hypertension, osteoporosis, schizophrenia, stroke, and substance use disorders.

### Covariates of interest

We selected patient population characteristics including sex, age group (18–39 years, 40–64 years, 65+ years), and race/ethnicity (White non-Hispanic, Black non-Hispanic, Hispanic, Asian Pacific Islander non-Hispanic, and Other). Race/ethnicity measures may be problematic in hospital discharge databases because some states do not collect information on race and ethnicity from hospitals and, within states that collect the information, some hospitals do not code race and ethnicity reliably. To deal with this problem, we used the SID Disparities Analysis File for 2012 designed for the AHRQ National Healthcare Disparities Report to provide national accurate estimates of race and ethnicity. A measure for race/ethnicity in the SID is created using a stratified, weighted sample of hospitals with good reporting of patient race and ethnicity from 38 SID states: Alaska, Arizona, Arkansas, California, Colorado, Connecticut, Florida, Georgia, Hawaii, Illinois, Indiana, Iowa, Kansas, Kentucky, Maryland, Massachusetts, Michigan, Missouri, Nevada, New Jersey, New Mexico, New York, North Carolina, Ohio, Oklahoma, Oregon, Pennsylvania, Rhode Island, South Carolina, South Dakota, Tennessee, Texas, Utah, Vermont, Virginia, Washington, Wisconsin, and Wyoming. It is drawn as a hospital sample of 40 % of community, nonrehabilitation hospitals in the United States (about 2000 hospitals) [19].

Additional patient circumstances explored in descriptive analyses were patient location (largest locales [metropolitan and micropolitan] and smallest locales [nonmetropolitan and nonmicropolitan]) and primary expected source of payment (private insurance, Medicare, Medicaid, other insurance, uninsured [includes uninsured, self-pay, no charge, and other])

### Analytic methods

All analyses used discharge-level data rather than hospital-level aggregates. Descriptive statistics were computed by tabulating the number and percentage of discharges observed in categories of MCCs and in categories of demographic characteristics of the sample for each set of ambulatory care sensitive conditions (i.e., within each PQI composite). We then developed multivariate models to examine associations between categories of MCCs and the three outcome variables: 1) inpatient costs per stay (dollars), 2) inpatient costs per day (dollars per day), and 3) length of inpatient hospitalization (days). As our data on costs per stay and per day was over-dispersed, we used a negative binomial 2 regression model to accommodate this skewed cost data. We used generalized linear models to model length of stay (LOS). Hospital costs were derived by converting reported charges data to estimated costs using hospital-specific cost-to-charge ratios contained in the 2012 HCUP SID Cost-to-Charge Ratio files (https://www.hcup-us.ahrq.gov/db/state/costtocharge.jsp#user).

All models included the primary independent variable, number of MCCs. Zero or 1 chronic condition was the reference category in multivariate analyses. We also controlled for the population characteristics using males, 65+ years of age, and White race/ethnicity as the reference categories.

Because inpatient mortality could confound the relationship between cost and MCCs, we adjusted models to assess whether findings were affected by discharge disposition. We used two indicators. The first indicator—dead or alive—was tested in the inpatient hospital cost per stay regression model. The second indicator used eight discharge dispositions—home or self-care, transfer to short-term hospital, transfer to other facility, home health care, against medical advice, died in hospital, discharged alive, and unknown or missing—to examine other potential reasons for the longer LOS for patients with MCCs.

## Results

### Descriptive results

A descriptive overview of the sample population characteristics is provided in Table 1. Over half of patients discharged in 2012 for ambulatory care sensitive conditions were 65 years or older. Likewise, Medicare was the most prevalent primary expected payer (64 %), followed by private insurance (16 %). The majority of patients were female (56 %), were White Non-Hispanic (69 %), and resided in a metropolitan area (91 %).

**Table 1:** Characteristics among inpatient hospital stays for ambulatory care sensitive conditions included in the AHRQ PQIs

Characteristic	Overall composite (PQI 90)	Acute composite (PQI91)	Chronic composite (PQI 92)
Discharges (n)			
Total (weighted)	3,580,785	1,402,097	2,178,765
Mean cost per discharge (USD)	$8085	$7551	$8431
Mean Length of Stay (days)	4.4	4.3	4.4
Discharges (%)			
Without MCC (0–1 chronic condition)	13.2	21.8	7.7
2–3 chronic conditions	33.7	37.8	31.0
4–5 chronic conditions	35.3	30.1	38.7
6+ chronic conditions	17.8	10.2	22.6
Age (%)			
18–39 years	8.9	8.9	8.9
40–64 years	33.2	26.7	37.5
65+ years	57.8	64.3	53.6
Sex (%)			
Male	43.7	39.2	46.6
Female	56.3	60.8	53.4
Race/ethnicity (%)			
White non-Hispanic	69.3	76.0	65.0
African American non-Hispanic	17.5	11.3	21.5
Hispanic (of any race)	9.2	8.7	9.5
API non-Hispanic	1.5	1.6	1.5
Other non-Hispanic	2.4	2.4	2.5
Patient location (%)			
Metropolitan; micropolitan	90.7	89.1	91.7
Non-metro-; Non-micropolitan	9.3	10.9	8.3
Primary expected payer (%)			
Private insurance	15.8	16.6	15.2
Medicare	63.8	67.2	61.6
Medicaid	11.4	8.8	13.0
Other insurance	2.5	2.2	2.7
Uninsured/self-pay/no charge	6.6	5.2	7.5
Discharge status			
Missing	0.02	0.02	0.02
Home or self-care	61.32	58.08	63.41
Short-term hospital	2.02	1.71	2.22
Other type of facility	17.28	22.29	14.06
Home health care	16.05	14.86	16.82
Against medical advice	1.61	1.06	1.97
Died in hospital	1.65	1.94	1.47
Alive; destination unknown	0.04	0.04	0.04

*Abbreviations*: *API* Asian Pacific Islander, *MCC* multiple chronic condition, *PQI* Prevention Quality Indicator

Source: Agency for Healthcare Research and Quality, Healthcare Cost and Utilization Project, State Inpatient Databases, 2012

Among patients hospitalized for acute ambulatory care sensitive conditions in 2012, only 22 % did not have MCCs present (Table 1). The most common category observed was 2 or 3 chronic conditions (38 % of patients), followed closely by those with 4 or 5 chronic conditions (30 % of patients); 10 % of patients had 6 or more chronic conditions. Patients who were hospitalized for a potentially preventable chronic condition had a higher number of MCCs than those with acute conditions. The third category (4 or 5 chronic conditions) was the most prevalent (39 % of stays), followed by the 2 or 3 chronic-condition category (31 % of stays). The highest category of MCCs (6+ conditions) represented 23 % of discharges among those hospitalized for an ambulatory care sensitive chronic condition.

### Multivariate regression results: costs

We examined inpatient hospitalization costs in relation to the number of MCCs present adjusting for age, sex, and race/ethnicity (Table 2). Compared with patients with 0 or 1 chronic condition, total hospitalization costs for ambulatory care sensitive conditions were 19 % higher for those with 2 or 3 chronic conditions (95 % confidence interval [CI] 1.19–1.20), 32 % higher for those with 4 or 5 chronic conditions (95 % CI 1.31–1.32), and 31 % higher for those with 6 or more chronic conditions (95 % CI 1.30–1.32). We observed similar patterns per hospitalization for potentially preventable acute conditions: compared with patients with 0 or 1 chronic condition, relative costs were 14 % higher for those with 2 or 3 conditions (95 % CI 1.14–1.15), 26 % higher for those with 4 or 5 conditions (95 % CI 1.25–1.27), and 33 % higher for those with 6+ conditions (95 % CI 1.32–1.34). Among those hospitalized for potentially preventable chronic conditions, inpatient costs per stay were 23 % higher if 2 or 3 chronic conditions were present (95 % CI 1.22–1.24), 33 % higher if 4 or 5 chronic conditions were present (95 % CI 1.32–1.34), and 28 % higher if 6+ chronic conditions were present (95 % CI 1.27–1.29)., compared with 0 or 1 chronic condition. We adjusted models for mortality status at discharge and saw very slight attenuation of these associations (Table 3).

**Table 2:** Multivariable regressions of total inpatient hospital costs by MCCs category among ACSCs in the AHRQ PQIs

	Overall composite (PQI 90)		Acute composite (PQI 91)		Chronic composite (PQI 92)	
Total inpatient costs^a^	Relative costs	95 % CI	Relative costs	95 % CI	Relative costs	95 % CI
Intercept (dollars per day)	$6583		$6495		$6751	
0–1 chronic condition	1.00	Ref.	1.00	Ref.	1.00	Ref.
2–3 chronic conditions	1.19	(1.19–1.20)	1.14	(1.14–1.15)	1.23	(1.22–1.24)
4–5 chronic conditions	1.32	(1.31–1.32)	1.26	(1.25–1.27)	1.33	(1.32–1.34)
6+ chronic conditions	1.31	(1.30–1.32)	1.33	(1.32–1.34)	1.28	(1.27–1.29)

*Abbreviations*: *ACSC* ambulatory care sensitive condition, *CI* confidence interval, *MCC* multiple chronic condition, *PQI* Prevention Quality Indicator

^a^ Model adjusted for age, sex, race. Reference categories are 65+ years old, male, and white

Source: Agency for Healthcare Research and Quality, Healthcare Cost and Utilization Project, State Inpatient Databases, 2012

**Table 3:** Multivariable regressions of hospitalization costs per inpatient day and LOS by MCCs category among ACSCs in the AHRQ PQIs

	Overall composite (PQI 90)		Acute composite (PQI 91)		Chronic composite (PQI 92)	
Costs per inpatient day^a^	Relative costs	95 % CI	Relative costs	95 % CI	Relative costs	95 % CI
Intercept (dollars per day)	$1937		$1896		2057	
0–1 chronic condition	1.00	Ref.	1.00	Ref.	1.00	Ref.
2–3 chronic conditions	1.03	(1.03–1.03)	1.01	(1.00–1.01)	1.01	(1.01–1.02)
4–5 chronic conditions	1.05	(1.04–1.05)	1.01	(1.01–1.02)	1.02	(1.01–1.02)
6+ chronic conditions	1.04	(1.03–1.04)	1.01	(1.01–1.02)	0.99	(0.99–1.00)
Length of stay-Model 1^a^	Relative stay	95 % CI	Relative stay	95 % CI	Relative stay	95 % CI
Intercept (Days)	3.88		3.86		3.86	
0–1 chronic condition	1.00	Ref.	1.00	Ref.	1.00	Ref.
2–3 chronic conditions	1.13	(1.13–1.14)	1.11	(1.11–1.12)	1.17	(1.16–1.18)
4–5 chronic conditions	1.22	(1.22–1.23)	1.21	(1.20–1.22)	1.24	(1.23–1.25)
6+ chronic conditions	1.23	(1.22–1.23)	1.27	(1.26–1.28)	1.22	(1.21–1.23)
Length of stay-Model 2^b^	Relative stay	95 % CI	Relative stay	95 % CI	Relative stay	95 % CI
Intercept (Days)	2.80		2.96		2.77	
0–1 chronic condition	1.00	Ref.	1.00	Ref.	1.00	Ref.
2–3 chronic conditions	1.15	(1.15–1.16)	1.12	(1.11–1.13)	1.18	(1.17–1.20)
4–5 chronic conditions	1.24	(1.23–1.25)	1.20	(1.19–1.21)	1.24	(1.22–1.25)
6+ chronic conditions	1.22	(1.21–1.23)	1.25	(1.24–1.26)	1.18	(1.16–1.19)

*Abbreviations*: *ACSC* ambulatory care sensitive condition, *CI* confidence interval, *LOS* length of stay

^a^ Model adjusted for age, sex, race

^b^ Model adjusted for age, sex, race and discharge status

Source: Agency for Healthcare Research and Quality, Healthcare Cost and Utilization Project, State Inpatient Databases, 2012

### Multivariate regression results: cost per day and LOS

We used two measures to evaluate cost per stay: cost per day (intensity of daily care) and LOS (duration of care). We examined how the number of MCCs was related to cost per day and LOS of stay for inpatient care (Table 3). Among hospitalizations for both acute and chronic ambulatory care sensitive conditions, we did not observe meaningful associations between the presence of a higher number of MCCs and cost per day of hospitalization. In contrast, we did observe positive associations between the presence of more chronic conditions and longer stays in the hospital.

In the acute composite group, compared with those with 0 or 1 chronic condition, hospital stays were 11 % longer when 2 or 3 chronic conditions were present (95 % CI 1.11–1.12), 21 % longer when 4 or 5 conditions were present (95 % CI 1.20–1.22), and 27 % longer when 6+ conditions were present (95 % CI 1.26–1.28). Similarly, for the chronic composite group, the presence of 2 or 3 chronic conditions was associated with a 17 % longer stay (95 % CI 1.16–1.18), 4 or 5 chronic conditions was associated with a 24 % longer stay (95 % CI 1.23–1.25), and 6+ chronic conditions was associated with a 22 % longer stay (95 % CI 1.21–1.23) than those with just 1 chronic condition. Our additional adjustment for discharge status resulted in little change in the estimates, although some attenuation was noted for the 6+ condition category in both the acute and chronic composite groups (Table 3).

## Discussion

Multiple chronic conditions are highly prevalent among U.S. patients hospitalized for potentially preventable acute and chronic conditions. In this study, more than 90 % of those hospitalized for ambulatory care sensitive chronic conditions had 2 or more chronic conditions, and over 20 % had 6 or more chronic conditions. For those hospitalized for potentially preventable acute conditions, nearly 80 % had MCCs and more than 10 % had 6 or more chronic conditions. This study’s findings on prevalence are consistent with previously published literature on hospitalization trends for people with MCCs. Other studies have reported higher utilization among patients with more chronic conditions [6, 9, 20, 21]. A study of 2009 HCUP discharge data showed that two-thirds of hospitalized patients had MCCs [6]; by 2012 we found that 80–90 % of patients in our sample had MCCs. Other studies have shown increasing rates of MCCs in the United States [9].

The high prevalence of MCCs among hospital patients indicates that we need better understanding and treatment of these patients. What is the pattern of their use of health care services across the care spectrum? Will improving coordination of care, monitoring patient conditions, and directing patients to more appropriate health care settings result in better health outcomes and greater efficiencies of utilization? HHS has developed a strategic framework with goals of strengthening the U.S. health care and public health systems, empowering individuals to use self-care management, equipping health care providers with tools and interventions, and supporting targeted research on MCCs to create effective interventions [22]. For example, AHRQ has developed tools that physicians can use to enhance ambulatory care for patients by improving communication with patients, by encouraging patients to obtain preventive services and to schedule evaluations and treatments on time, and by monitoring patients for medication effectiveness and adverse events, among other evidence-based approaches [23].

Consistent with our inpatient cost results, other studies have shown higher inpatient health care costs for patients with MCCs [6, 11, 20]. Authors of a systematic literature review of 35 studies found that almost all studies showed a positive monotonic relationship between cost and MCCs [20]. For example, in a study on the impact of MCCs among the Medicare population, annual payment amounts per beneficiary for all settings of care and for those with only 1, 2, and 3 or more conditions were $7172, $14,931, and $32,498, respectively, in 2005 dollars [24]. In our study of inpatient costs for all payers in 2012, costs per stay for the reference category, white males 65 and older, were $6583, $7833, $8689, and $8623 for 0 or 1, 2 or 3, 4 or 5, and 6 or more conditions, respectively. In our study, the higher cost of inpatient treatment was driven by longer lengths of stays rather than by higher costs per day on average. This result suggests that patients admitted and initially treated for one condition may require extra days for monitoring and treatment of one or more of their secondary conditions. For example, a patient admitted with hypertension and diabetes might be managed quickly in terms of blood pressure, but not in terms of blood glucose. Achieving a reasonable glucose level may require extra days in the hospital. Diabetes management and control would not require large outlay costs, as a surgical intervention might, but they would require a longer stay for treatment, for titration of medication, and for development of a suitable post-discharge plan. Alternatively, the data may suggest that individuals that present to the hospital with multiple chronic conditions may have more severe complications with their primary condition upon arrival at the hospital that require greater lengths of stay. For example, an individual with diabetes and depression that is admitted to a hospital due to diabetic complications may, on average, have a more severe complication and require longer stays than, on average, an individual that is admitted to a hospital with diabetic complications but has no other chronic diseases. We note that the subgroup with 6 or more chronic conditions has essentially the same costs as those with 4 to 5 such conditions; the lack of a monotonically increasing cost for the 6+ group suggests that additional diagnostic coding, perhaps motivated by reimbursement, does not add information about risk related to the cost of a stay. Counting up to 5 or more chronic conditions may be sufficient for profiling the relationship of chronic conditions to utilization and outcomes.

One implication of our research is the potential value from incorporating MCCs or numbers of MCCs into risk adjustment, quality and performance measurement, and other analytics. The statistically significant findings that more MCCs result in longer hospital stays and higher costs per stay suggest that the number of MCCs, if not specific MCC combinations, should be analyzed whenever severity of a patient’s condition is a potential contributing factor to an outcome or a potential distinguishing feature of a population. The number of *chronic* coexisting conditions typically is not used as a measure of severity in health services research or performance measurement, although particular comorbidities may be specified in various risk adjustment schemes. Findings from this study suggest that the number of MCCs could be a simple and useful predictor of utilization and costs of health care services that account for the complexity of patients’ conditions. Furthermore, health policymakers might test the effect of number of MCCs on reimbursement formulas and provider performance measures to create equitable comparisons and adjustments of payments among providers.

Our study benefited from the use of the HCUP SID, a large discharge database of experience for all payers. Use of the PQI conditions facilitated examining MCCs for patients who share potentially preventable conditions. Despite these strengths, our results should be interpreted in light of their limitations. First, only a portion of the total costs of care for chronic disease can be studied using hospital data. Although hospital stays account for a large portion of the cost of some chronic conditions such as heart failure, they do not include office visits, clinical tests, drug therapy, and other ancillaries. Second, only 38 of the 50 states provided data that could be used for this study, so the study is not nationally representative, despite over 3.5 million discharges for the 12 conditions reflected in the composite PQIs.. Third, variation in the presence of MCCs reflects only a portion of the variability of hospital costs among patients in our analysis. In particular, we did not address other patient, hospital, and community factors that influence costs. Our intent for this analysis was to explore the value of future work on MCCs, which we confirmed. Fourth, enumerating the number of chronic conditions present in hospital records depends on the extent of coding of secondary diagnoses in the data. To the extent that recording may be truncated, the number of MCCs may have been underestimated. In addition, our use of an enumeration of chronic diseases, rather than incorporating measures of the severity of an individual’s illness, such as the Charlson co-morbidity index [25], or a consideration of each individuals specific disease combinations based on their specific disease profile, as done by Kadam and colleagues [26], prohibits us from identifying which combinations of diseases are most strongly associated with the outcomes of interest. However, the enumeration and categorization of conditions aligns our results with operating definitions of MCC outlined by the United States Department of Health and Human Services initiatives that underlie research and policy considerations on this topic and does allow for a general understanding of MCCs that would not be possible considering disease combinations in isolation.

## Conclusions

In this large sample of records of U.S. hospitalizations, we observed a positive association between the presence of MCCs and hospitalization costs for potentially preventable conditions. The higher costs associated with MCCs was driven by longer hospital stays rather than by higher costs per day. To our knowledge, this is the first study to examine MCC in relation to costs and length of stay for hospitalizations that are potentially preventable through high-quality ambulatory care. e. When considering trends in costs and utilization related to preventable hospitalizations, studies have indicated that the occurrence of preventable hospitalizations is decreasing in the United States [8]. However, it is important to consider that at the same time, the prevalence of patients presenting with MCCs is increasing [6, 8]. The higher costs and utilization associated with these more complicated patients bring a greater burden with each preventable hospitalization. Thus, although the number of preventable hospitalizations is declining, our analyses indicate that some of the benefit anticipated with that reduction may be offset by an increase in costs and utilization driven by a rise in the prevalence of MCCs. Patients with MCCs are a challenge to treat because of the number, complexity, and interaction of their illnesses. Patients with a high number of morbidities may be experiencing end-of-life crises that lead them to the hospital repeatedly; recent insights on how the medical profession deals with patients at the end of life suggest that the health care system could benefit from educating clinicians on how to deal with these situations realistically and humanely [27].

In this study we specifically examined the hospital experience, but treatment spans all settings of care. A greater understanding of how MCCs influence care costs and utilization from ambulatory care through the inpatient setting may help providers across this spectrum identify ways to work together to achieve optimum health outcomes for their patients and to reduce hospitalization costs [12].

## Abbreviations

AHRQ: Agency for healthcare research and quality
API: Asian Pacific Islander
CI: Confidence intervals
HCUP: Healthcare cost and utilization project
HHS: U.S. Department of Health and Human Services
LOS: Length of stay
MCC: Multiple chronic condition
PQI: Prevention quality indicators
SID: State inpatient databases
